# Alemtuzumab for Multiple Sclerosis

**DOI:** 10.1007/s11910-016-0685-y

**Published:** 2016-08-02

**Authors:** Mark D. Willis, Neil P. Robertson

**Affiliations:** Institute of Psychological Medicine and Clinical Neuroscience, University Hospital of Wales, Cardiff University, Heath Park, Cardiff, CF14 4XN UK

**Keywords:** Multiple sclerosis, Alemtuzumab, Autoimmunity

## Abstract

Alemtuzumab is a humanised anti-CD52 monoclonal antibody approved for use in active, relapsing multiple sclerosis (MS). Administration results in a rapid depletion of circulating lymphocytes with a subsequent beneficial immune reconstitution. Early open-label experience and recent clinical trials have demonstrated a dramatic effect on relapse rates as well as a positive effect on radiological disease outcomes and disability measures. Despite a mechanism of action that results in profound lymphopaenia, opportunistic infections are rarely seen and no excess association with malignancy has been identified. However, acquired autoimmune disease (AID) is a common adverse event following treatment, necessitating rigorous monitoring in order to facilitate prompt detection and management. Despite this issue, a unique dosing schedule and durability of effect make alemtuzumab a welcome addition to currently available treatment options for MS.

## Introduction

Multiple sclerosis (MS) is a common, inflammatory disease of the central nervous system, responsible for substantial morbidity in western populations [1]. The underlying pathology is complex and remains poorly understood. However, amongst a complex interplay of immunological factors, T lymphocytes are known to occupy a central role in disease pathogenesis [2, 3]. Over the last two decades, an increased understanding in disease biology and genetic susceptibility has contributed to the development of novel therapeutic interventions which are largely designed to target function and migration of T lymphocytes [4]. With a substantial therapeutic armamentarium now available, it has become imperative to understand the clinical effects of these interventions and their place in treatment regimes.

Alemtuzumab, an anti-CD52 monoclonal antibody, was first proposed as a treatment for MS in the 1990s [5, 6]. Following clinical trials demonstrating a dramatic effect on relapse rates, in addition to a positive effect on longer term disability outcomes [7–9], it has now been approved for use in 49 countries worldwide [10]. Its primary indication is for active relapsing disease, either as first- or second-line treatment, although in a small number of countries has been restricted to patients who have had an inadequate response to two or more established disease-modifying therapies. As well as having an impressive clinical effect across a number of end points including relapse rate, brain atrophy and measures of disability, treatment with alemtuzumab has also offered some fascinating insights [11, 12] into clinical aspects of MS and allowed a greater understanding of disease pathogenesis. Furthermore, as a result of the now well-established side effect of disease-specific autoimmunity, it has provided an unintended but intriguing window into the origins of human autoimmune disease.

In this review article, we aim to summarise the mechanism of action of alemtuzumab and key findings from clinical trials as well as more recently published long-term follow-up data. We will also outline the main side effects of this therapy and offer practical points for its use in clinic practice.

## Mechanism of Action

Alemtuzumab is a humanised monoclonal antibody targeted against the cell surface protein CD52 [13]. The function of this molecule is largely unknown, although is thought to contribute to T cell activation [14], migration [15] and the induction of regulatory T cells [15]. CD52 is present primarily on the cell surface of lymphocytes but also at lower levels on monocytes, macrophages, eosinophils and NK cells [16–18]. Epithelial cells of the epididymis, vas deferens and seminal vesicle also express CD52 at low levels [16–18]. However, of critical Importance, a lack of CD52 expression on bone marrow-derived haematopoietic precursors allows lymphocyte reconstitution following treatment and return of immune competency [19].

**Fig. 1:**
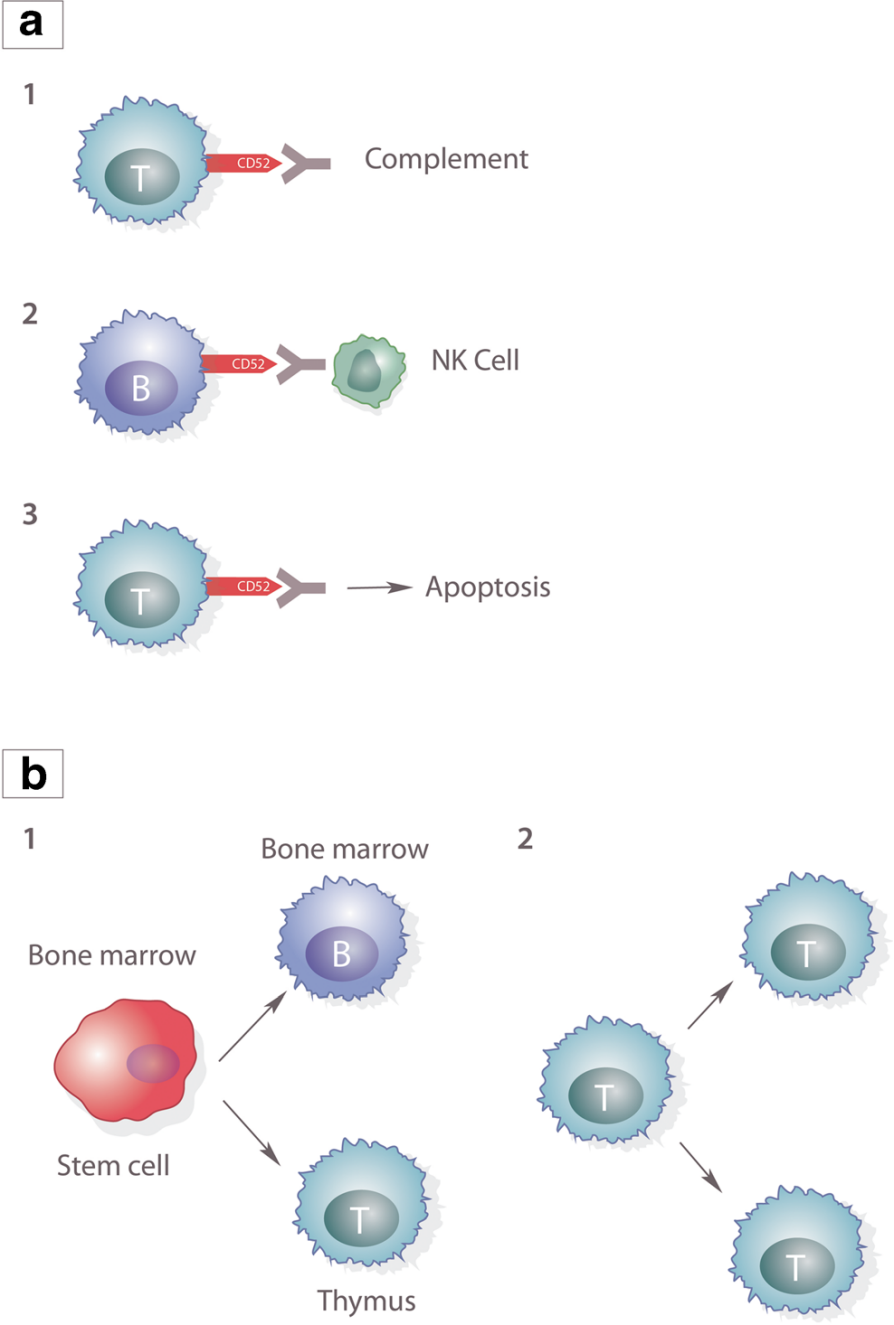
Proposed mechanism of action of alemtuzumab. **a** Alemtuzumab targets B and T lymphocytes by one of three mechanisms. *1* Complement-mediated cytolysis. *2* Antibody-dependent cytolysis. *3* Induction of apoptosis. **b** Lymphocyte repopulation occurs through either *1* production of new T and B lymphocytes or *2* through homeostatic repopulation of surviving lymphocytes

Alemtuzumab treatment results in a rapid and profound reduction in peripheral lymphocytes due to antibody-dependent cell-mediated cytotoxicity [20], complement-dependent cytolysis and induction of apoptosis [21] with a subsequent beneficial reconstitution of the immune system [22]. Repopulation occurs via two mechanisms: proliferation of mature lymphocytes that escape deletion (‘homeostatic proliferation’) and via bone marrow/thymic repopulation (Fig. 1).

Following treatment, rates of lymphocyte recovery vary by cell type, with B lymphocytes first to recover followed by CD8+ and CD4+ T lymphocytes [23–25]. Although controversial, the rate and pattern of lymphocyte reconstitution are not currently thought to correlate with subsequent re-emergence of disease activity [23, 26, 27]. As immune reconstitution becomes more established, regulatory CD4+ T cells (Tregs) dominate the T cell population and are considered to be one of the factors contributing to long-term efficacy rather than this being solely a result of lymphodepletion [28–30]. In particular, a recent study reporting results from the phase III trials has demonstrated a significant increase in Treg cell percentage at 24 months after treatment [31]. An increased representation of memory T lymphocytes is also observed [32], although the impact of this phenomenon is less clear. Furthermore, mRNA levels of pro-inflammatory cytokines and anti-inflammatory cytokines are downregulated and upregulated, respectively, following treatment, which may also contribute to the drug’s unique durability in MS [31].

The potential role of neutralising antibodies to alemtuzumab (which have been identified following treatment and become less frequent in subsequent courses) in modifying efficacy remains unclear. However, levels can be reduced by co-administration of SM3, a non-cell binding variant of alemtuzumab [33], which, whilst not currently available for routine clinical use, may offer a future route for managing a subset of patients who fail treatment because of the development of neutralising antibodies.

## Development of Alemtuzumab in the Treatment of MS

### Early Experience

Prior to its use as a therapy for MS, alemtuzumab was licensed for fludarabine resistant chronic lymphocytic leukaemia in addition to its application in organ transplantation and other autoimmune disorders [34]. Early in the clinical development programme for MS, alemtuzumab was used in patients with advanced progressive disease. Although radiological outcomes were encouraging, disability accumulation continued with increased cerebral atrophy 7 years after treatment [5, 6, 11, 34]. In contrast, patients with relapsing disease experienced a reduction in annualised relapse rates (ARR) and an improvement in disability. This dichotomy of clinical outcomes between patients treated at an earlier stage of disease and those with progressive disease offered important insights into disease pathogenesis and timing of interventions. Early disease was concluded to be the result of a more active inflammatory demyelinating phase and followed by a later phase of axonal degeneration and accumulation of disability. Subsequent investigation therefore focused on the inflammatory disease subtype characterised clinically by a relapse dominant disease course, with two open-label trials in treatment-naïve and treatment refractory patients showing encouraging clinical outcomes [35, 36].

### Clinical Trials (CAMMS223, CARE-MSI and CARE-MSII)

One phase II (CAMMS223) [9] and two phase III (Comparison of Alemtuzumab and Rebif® Efficacy in Multiple Sclerosis (CARE-MS) I and II) [7, 8] clinical trials were undertaken following these positive early experiences. CAMMS223 compared low- and high-dose alemtuzumab against a high-dose active comparator (subcutaneous interferon beta 1-a, Rebif®, 44 μg three times weekly) in patients with early, active, relapsing-remitting MS [9]. CARE-MSI [7] and CARE-MSII [8] investigated the use of alemtuzumab in treatment-naïve patients and in patients previously on disease-modifying therapy who had experienced an inadequate response (≥1 relapse), respectively. As with the phase II study, interferon beta 1-a was used as an active comparator. Inclusion criteria and clinical outcomes for these trials are summarised in Table 1.

**Table 1:** Clinical outcomes of alemtuzumab-treated patients in phase II and phase III studies [37]

	CAMMS223 [9]	CARE-MSI (treatment-naïve) [7]	CARE-MSII (previous treatment) [8]
	All patients		12-mg group only
Number of alemtuzumab-treated patients	222	376	426
Inclusion criteria	Active RRMS (≥2 relapses in the prior 2 years and ≥1 Gd-positive lesion); treatment-naïve; EDSS ≤3; onset ≤3 years	Active RRMS (≥2 relapses in the prior 2 years and ≥1 relapse in the prior year); treatment-naïve; EDSS ≤3; onset ≤5 years	Active RRMS (≥2 relapses in the prior 2 years and ≥1 relapse in the prior year); relapsing on prior DMT; EDSS ≤5; onset ≤10 years
Follow-up (years)	3	2	2
Relapse rate reduction (alemtuzumab vs. interferon beta-1a)	74 % (*p* < 0.001)	55 % (*p* < 0.0001)	49 % (*p* < 0.0001)
Annualised relapse rate (alemtuzumab vs. interferon beta-1a)	0.10 vs. 0.36	0.18 vs. 0.39	0.26 vs. 0.52
% patients with 6-month SAD	9 vs. 26 % (*p* < 0.01)	8 vs. 11 % (not significant)	13 vs. 21 % (*p* < 0.01)
Change in mean EDSS from baseline	Improvement of 0.39 compared with deterioration of 0.38 on interferon beta-1a (*p* < 0.01)	No significant change	Improvement of 0.17 compared with deterioration of 0.24 on interferon beta-1a (*p* < 0.0001)
Deaths	1 (ITP), 1 (myocardial infarction)	1 (RTA)	1 (RTA), 1 (aspiration pneumonia)
Autoimmunity			
Thyroid	26 %	18 %	17 %
ITP	0.9 %	0.8 %	1 %
Goodpasture’s syndrome	0	1	0
Neoplasia (alemtuzumab vs. interferon beta-1a)	2.8 vs. 0.9 %	0.5 vs. 0 %	0.6 vs. 1.5 %

Adapted from Coles [38]

*SAD* sustained accumulation of disability, *RTA* road traffic accident, *EDSS* expanded disability status score, *ITP* idiopathic thrombocytopenic purpura, *DMT* disease-modifying therapy, *Gd* gadolinium, *RRMS* relapsing-remitting MS

### CAMMS223

Treatment-naïve patients (334) with a diagnosis of relapsing-remitting MS (RRMS) were randomised to alemtuzumab 12 mg/day, alemtuzumab 24 mg/day or high-dose subcutaneous interferon beta 1-a three times weekly. Results from this study were impressive both for clinical and radiological outcomes. The pooled (12 and 24 mg) alemtuzumab groups demonstrated a reduction in ARR of 74 %, reduction in sustained accumulation of disability (SAD; a ≥1-point increase in Expanded Disability Status Score (EDSS) [39] from baseline if baseline EDSS >0, or ≥1.5 point increase if baseline EDSS = 0, persistent over a 6-month period) of 71 % and improvement in mean EDSS score of 0.39 points at 36 months. In contrast, patients treated with interferon beta 1-a experienced a worsening of EDSS score of 0.38 points over the same time period. Radiologically, reduction in brain volume was significantly less in the pooled alemtuzumab treatment group. Similarly, although reduction in lesion volume on T2-weighted MRI was seen in both alemtuzumab and beta interferon patients, this was more notable in the alemtuzumab groups, with significance seen at 12 and 24 months; however, at 36 months, this effect was not significant [9].

The cohort of patients involved in CAMMS223 continued to demonstrate improvements in EDSS at 5 years of follow-up, although the majority of this effect was in the first 36 months [40]. A post hoc analysis using a new disability outcome, sustained reduction of disability (SRD, a reduction from baseline of at least 1 EDSS point confirmed over 6 months for patients with a baseline EDSS ≥2.0), demonstrated more alemtuzumab-treated patients achieved this outcome compared with interferon-treated patients [41].

### CARE-MSI and CARE-MSII

In the phase III follow-up to CAMMS223, CARE-MSI and CARE-MSII investigated alemtuzumab therapy in treatment-naïve and treatment-experienced patients, respectively. These studies were conducted over a 2-year period with the primary endpoints of ARR and time to 6-month SAD [7, 8]. In CARE-MSI, patients received alemtuzumab at a dose of 12 mg/day [7]. In CARE-MSII alemtuzumab, patients were randomised to a dose of either 12 or 24 mg/day, although after 1 year of the study, all patients received 12 mg [8]. Discontinuation of randomization to the 24-mg/day group was undertaken because of safety concerns following the reported case of ITP but also to aid recruitment to the remaining study groups.

Once again, alemtuzumab demonstrated superiority to interferon beta 1-a. Patients experienced a reduction of ARR in CARE-MSI and CARE-MSII by 55 and 49 %, respectively [7, 8]. EDSS score was also improved in the alemtuzumab groups in both studies. Although this was significant in CARE-MSII (improvement of 0.17 points on alemtuzumab vs. a worsening of 0.24 in the interferon beta 1-a group), both groups experienced an improvement in EDSS in CARE-MSI (improvement of 0.14 points in both groups), which did not achieve significance [7, 8]. Similarly, in CARE-MSII, significantly fewer patients had SAD (13 vs. 20 %) and more patients had SRD (22 vs. 9 %) in the alemtuzumab group. Again, in contrast to CARE-MSII, significance was not achieved in SAD in CARE-MSI, although SRD was not measured [7, 8].

Interim data from the ongoing phase III extension study have demonstrated marked durability over 5 years [42, 43]. For patients enrolled in CARE-MSI and CARE-MSII, the low ARR was maintained in year 3 (0.19 and 0.22, respectively) to year 5 (0.15 and 0.18). For years 0–5, 80 % patients in CARE-MSI and 75 % patients in CARE-MSII were free from 6-month SAD. Impressively, 69 and 65 % patients, respectively, had stable/improved EDSS scores and 33 and 43 % patients experienced SRD in years 0–5 [42, 43]. It has been suggested that the improvement in disability observed following treatment might be as a result of increased lymphocytic delivery of neurotrophins to the CNS aiding neuroprotection [44].

Radiological outcomes were also significantly better in the alemtuzumab-treated patients compared with interferon beta 1-a. In particular, change in brain volume (BV), gadolinium-enhancing lesions and patients with new or enlarging T2 hyperintense lesions on MRI were significantly better in the alemtuzumab groups in both studies [7, 8]. In the recently presented data from the extension study, these radiological changes also appear to have durability after 5 years of follow-up. Median rate of BV loss decreased progressively over 4 years in CARE-MSI and remained low in year 5 (year 1, −0.59 %; year 2, −0.25 %; year 3, −0.19 %; year 4, −0.15 %; year 5, −0.20 %). Similarly, median rate of BV loss progressively slowed over 3 years in CARE-MS II and remained low in years 4 and 5 (year 1, −0.48 %; year 2, −0.22 %; year 3, −0.10 %; year 4, −0.19 %; year 5, −0.07 %). Strikingly, the majority of patients (68 % in CARE-MSI and 60 % in CARE-MSII) had not received further courses of alemtuzumab treatment since month 12 [45]. Durability of MRI outcomes has also been shown in the extension study with respect to gadolinium (Gd)-enhancing lesions, new/enlarging T2 or new T1 lesions. In years 3, 4 and 5 after initial treatment, the proportion of patients free of the aforementioned measures was similar to those in year 2 (i.e. the end of the original phase III studies). In addition, most patients were free of MRI activity in each of years 3, 4 and 5 [46, 47].

### Open-Label Cohorts

In recent years, long-term follow-up data from open-label cohorts have also been available (Table 2). After a median 7-year follow-up of 87 patients with RRMS from two previous studies, 6-month SAD and SRD were observed in 32.2 and 43.5 %, respectively, in patients treated by a group in Cambridge, UK. Area under the curve analysis demonstrated an overall improvement or stabilisation of disability in 59.8 % patients. Consistent with previous studies, ARR was drastically reduced by 91 %. Mean EDSS reduction was 0.2 points [48]. Further data from this cohort has recently been presented: after a median time of 10.1 years, 45 % patients with RRMS remained relapse-free. Forty-one patients (48 %) required the standard two cycles of alemtuzumab, whilst relapses triggered re-treatment to a total of three cycles (in 33 patients (38 %)), four cycles (in nine patients (10 %)) and five cycles (in three patients (3 %)). Disability measured by EDSS remained largely unchanged. Conversely, for 36 patients with secondary progressive disease (with a median follow-up of 19.8 years), the median EDSS significantly increased from 6 to 8.75 [50].

**Table 2:** Clinical outcomes and adverse events of alemtuzumab-treated patients in two open-label cohorts [37]

	Cambridge open-label long-term follow-up cohort [48]	Cardiff regional cohort [49]
Patient number	87	100
Follow-up (years)	7 (median)	6.1 (mean)
Relapse rate reduction from baseline	91 %	90 %
% patients with 6-month SAD	32 %	27 %
Change in mean EDSS from baseline	−0.2	+0.14
Autoimmunity		
Thyroid	41 %	35 %
ITP	3.4 %	3 %
Goodpasture’s syndrome	1 %	0 %

*SAD* sustained accumulation of disability, *EDSS* expanded disability status score, *ITP* Idiopathic thrombocytopaenic purpura

In our own cohort encompassing 100 patients treated in Bristol and South Wales, UK, we observed similar reduction in ARR of 90 %. Forty patients underwent at least one additional course of treatment triggered by clinical or radiological changes. Although a reduction in mean EDSS was not observed, an increase of 0.14 EDSS points might still be considered a striking outcome since treatment had been reserved for patients with the most aggressive forms of disease [49].

## Adverse Events

The main adverse events following alemtuzumab include well-recognised infusion reactions, infections and acquired autoimmune disease including thyroid disorders, idiopathic thrombocytopaenic purpura (ITP) and immune-mediated nephropathies. Other rarely occurring autoimmune disease observed in the CARE-MS trials included neutropaenia, haemolytic anaemia, agranulocytosis and pancytopenia [7, 8].

### Autoimmune Disease

A unique observation in the use of alemtuzumab in MS is the occurrence of acquired autoimmune disease (AID), which is not observed in other conditions treated with this drug [51]. A range of autoimmune conditions has been described and in our own long-term follow-up cohort amounted to 47 % of patients [49]. Autoimmune disease of the thyroid gland is the most commonly occurring condition, with others including ITP and anti-glomerular basement membrane (anti-GBM) disease seen less frequently [7–9]. This development of novel autoimmunity is currently the focus of active research, offering a window into the genesis of human autoimmune disease. Whilst the underlying mechanism is still not fully understood, it is thought to occur as a result of subsequent homeostatic T cell proliferation following lymphodepletion [52], and where homeostatic proliferation predominates over thymic reconstitution, autoimmune disease is more likely [52]. Of interest, thyroid AID following alemtuzumab treatment is not seen in patients treated with B cell chronic lymphocytic leukaemia despite being given at higher doses suggesting a disease-specific phenomenon [51].

Early experience suggested an occurrence of thyroid AID in approximately 30 % of patients [34, 53]. Similar rates were seen in CAMMS223, CARE-MSI and CARE-MSII at 23, 18 and 16 %, respectively [7–9]. A large multi-centre cohort subsequently demonstrated thyroid AID rates of 17 % [54]. In recently published long-term follow-up cohorts, total AID specific to the thyroid has been shown to occur at a higher rate and probably reflects longer follow-up. In our own multi-centre cohort, 47 % developed AID of which 35 % were thyroid [49]. Similarly, in the Cambridge cohort, 48 % patients experienced any AID with thyroid AID occurring in 41 % of patients [48]. The incidence of thyroid AID appears to be greatest in the first 3 years following the initial treatment course [42, 43, 48, 49] and responds to conventional therapies and management [51]. Importantly, the risk of developing AID has been shown to be unaffected by the cumulative dose, dosage interval or dosage frequency [48, 54].

A variety of other autoimmune conditions have also been documented [49]. The most significant of which is ITP. CAMMS223 was suspended after three patients developed ITP with one patient dying of an intracerebral haemorrhage before a diagnosis could be made. During the suspension period, three further patients were diagnosed. In the subsequent phase III and open-label cohorts, the rate of ITP has been shown to be between 1 and 3 % [7, 8, 48, 49, 54]. ITP associated with alemtuzumab therapy is thought to be characterised by delayed presentation after drug exposure, responsiveness to conventional therapies (corticosteroids, intravenous IgG, anti-Rh(D), platelet transfusion, rituximab) and prolonged remission [55].

Although even less common, renal disease has been reported in four patients within the clinical trials—one case of anti-glomerular basement membrane (anti-GBM) disease and three cases of membranous nephropathy (one of which also had low-level anti-GBM antibodies). Each patient responded to prompt medical treatment with plasmapharesis, steroids and cyclophosphamide and diuretics for anti-GBM disease and membranous nephropathy, respectively [56]. Three further patients treated outside of the clinical trials have also developed anti-GBM disease. Despite aggressive immunotherapy, two patients became dialysis-dependent and ultimately required renal transplantation, whilst the other although not requiring renal replacement has end-stage renal disease (A Coles, personal communication). Higher levels of Il-21 have been shown to be associated with developing autoimmune disease post-alemtuzumab [57], but a test for this has so far not reached standard clinical practice [58].

### Infections

For a therapy whose main biological effect is lymphopaenia, rates of serious opportunistic infections might reasonably be expected to be high. In the CARE-MS studies, mild to moderate infections were seen in 67–77 % patients receiving the 12-mg dose of alemtuzumab (vs. 45 and 66 % of those receiving interferon β1a). Upper respiratory tract, urinary tract and herpes simplex and zoster virus infections remain the most commonly observed [7, 8, 48, 49]. Infections attributable to immunosuppression in open-label studies include spirochaetal gingivitis, pyogenic granuloma and listeria meningitis. In addition, one further case of listeria meningitis was observed in the CAMMS223 study [9] with two more cases also recently being reported [59].

Although it is clear that vigilance needs to be maintained for opportunistic infections which can be severe, the relatively low frequency given the mode of action of alemtuzumab is intriguing and likely to represent a relative sparing of cells of the innate immune system in addition to haemopoetic precursor cells in the bone marrow and thymus. Subsequent lymphocyte repopulation following treatment also aids immune surveillance, and murine models have demonstrated that the function of the remaining lymphocytes following treatment is unimpeded [60].

### Malignancy

Although malignancies were seen in the clinical trials, the studies were not powered to detect changes between the groups. In CARE-MSI and CARE-MSII, malignancies were observed in 0.5 vs. 0 % and 0.6 vs. 1.5 % in the 12-mg alemtuzumab and interferon beta-1a-treated patients, respectively [7, 8]. Of 1486 total patients treated with alemtuzumab in the clinical development programme, 29 (2 %) have developed neoplastic disease. Thyroid cancer accounted for six cases, which may relate to the increased thyroid surveillance undertaken in treated patients [61, 62]. All were stage 1 papillary type carcinomas and discovered 10–41 months after last dose. One patient had a preexisting lymph node at baseline that was subsequently found to contain papillary thyroid cancer [62]. Basal cell carcinoma (six patients), breast cancer (five) and malignant melanoma (four) occurred at a frequency >1 %.

Outside of clinical trials, one further case of papillary thyroid carcinoma [63], one case of malignant melanoma [64] and one case of Castleman’s disease (a prelymphomatous condition) [38] have separately been reported. In addition, in our recent long-term follow-up cohort of 100 patients, 10 patients developed malignant/pre-malignant conditions. Three patients had monoclonal gammopathy of uncertain significance (MGUS), one of which was also diagnosed with a meningioma, two basal cell carcinomas and five patients had cervical dysplasia. Despite the theoretical risk of cervical malignancy following T lymphocyte depletion, there is currently no definitive available evidence that individual risk is increased compared to that of the background population. Despite this, it is recommended that female patients undergo annual cervical screening following treatment [65]. It should be noted that the risk of malignancy following immunosuppression may take many years to manifest and therefore detailed post-marketing surveillance will be essential to better define long-term risks.

### Pregnancy

Alemtuzumab may cross the placental barrier and therefore potentially produce harmful effects on the foetus. In animal studies, reproductive toxicity has been demonstrated although data in humans is lacking. It is unknown therefore whether alemtuzumab administration effects reproductive capacity or teratogenicity [65]. Because of the theoretical risk of pregnancy adverse events, the parent company recommends effective contraception during and for 4 months following treatment. The most recently reported data of 1486 patients (64.8 % female) treated in the clinical trials reveals 179 pregnancies occurring in 131 patients. Of completed pregnancies, 104 (66 %) were live births. No congenital abnormalities have been observed in delivered infants. Excluding pregnancies with unknown outcomes, there were 36 (21 %) spontaneous abortions, 16 (9 %) elective abortions and 1 (0.6 %) stillbirth. Rates of spontaneous abortions are similar to those in the general population [66]. Although CD52 is expressed in the epididymis, seminal vesicle, sperm and seminal fluid, suggesting a theoretical risk of reduced male fertility, a small study has not demonstrated any effects on sperm motility, morphology or count [67].

## Practical Considerations

In the context of MS treatment, alemtuzumab is given intravenously at a dose of 12 mg/day for 5 consecutive days, followed 12 months later by a further 3-day course. Cytokine release following alemtuzumab treatment is now well recognised with a rise in TNF-α, IL-6 and IFN-γ accompanying lymphodepletion [12]. This phenomenon is thought to explain the transient worsening of symptoms experienced by patients treated at an early stage of the drugs use. This is thought to occur because of a direct action on partially demyelinated pathways and is mitigated by the use of concomitant intravenous methylprednisoloine for the first 3 days of any treatment cycle [12]. Other common side effects seen during infusion include headaches, rigors, pyrexia and rash. As such, anti-pyretics and anti-histamines are given on an ‘as required’ basis for symptomatic control. In the clinical trials, infusion reactions occurred in ≥90 % patients [7–9].

Because of the relative increased incidence of herpes infections following treatment, administration of oral acyclovir (200 mg BD) is recommended during and for 1 month following treatment [65], which has been demonstrated to reduce the risk of herpetic virus infection [68]. In addition, patients without a history of chickenpox or vaccination against varicella zoster virus (VZV) should be checked for anti-VZV antibodies and be considered for VZV vaccination >6 weeks prior to treatment [65]. Although a small study has demonstrated that humoral responses after alemtuzumab treatment to common inactive vaccines are still effective [69], there is no data available for the safety of live vaccination following treatment [65]. It is therefore recommended that patients should complete local immunisation requirements at >6 weeks before commencing treatment and do not receive live vaccinations following treatment [65]. Patients are also given dietary advice to avoid foodstuffs that may contain listeria including soft cheeses, pates and smoked seafood.

With a high incidence of adverse autoimmune events, a rigorous safety-monitoring programme is required (Table 3). Prior to treatment initiation, complete blood count, serum creatinine, thyroid function and urinalysis should be performed and on a monthly basis thereafter or three monthly for thyroid function tests. Monitoring should continue for 48 months following the last treatment course [65], and any abnormality in monitoring outcomes should prompt urgent referral to the appropriate specialty for management. Our own experience is that patient education with regards to identifying early symptoms of AIDs is of paramount importance in order that potentially serious conditions can be promptly identified. Because of the theoretical increased risk of cervical malignancy due to human papillomavirus (HPV), patients are also recommended to undergo cervical screening prior to treatment initiation and at annual intervals thereafter [65].

**Table 3:** Monitoring guidelines [37]

Monitoring test	Monitoring interval	Duration
Complete blood count	Prior to treatment initiation and at monthly intervals thereafter	For 48 months following last treatment course
Serum creatinine	Prior to treatment initiation and at monthly intervals thereafter	For 48 months following last treatment course
Urinalysis with microscopy	Prior to treatment initiation and at monthly intervals thereafter	For 48 months following last treatment course
Thyroid function tests	Prior to treatment initiation and every 3 months thereafter	For 48 months following last treatment course

Within the clinical trials, patients were retreated with a further 3-day course of alemtuzumab on the basis of ≥1 protocol defined relapse or ≥2 new or Gd-enhancing or enlarging brain or spinal cord lesions. Recently, presented data has shown that 68 and 50 % of patients have received no treatment since month 12 of CARE-MSI and CARE-MSII, respectively [42, 43]. In our practice, patients have been retreated either for (i) clinical relapse only, (ii) new radiological lesions (with or without enhancement), (iii) clinical relapse and new radiological lesions, (iv) worsening disability and new radiological lesions or (v) worsening disability without change in EDSS [49]. Twenty-eight percent of patients have received three treatments, 11 % four treatments and one patient five treatments [49]. However, there is no current consensus on retreatment thresholds and further trials are needed for clarification.

## Conclusions

With options for disease-modifying therapies (DMTs) in MS continuing to expand, the question of when and how best to utilise the available intervention is becoming ever more relevant [70]. Alemtuzumab has been shown to have a dramatic effect on relapse rates in patients with MS as well as a positive effect on disability and radiological outcomes. However, it remains unclear whether alemtuzumab should predominantly be reserved for patients who fail standard DMTs such is the case in the USA or as a first-line option to treat selected patient sub-groups during a putative ‘window of therapeutic opportunity’. So-called escalation and induction regimes are the focus of substantial recent conjecture with clinical trials urgently needed to help resolve this issue. As well as efficacy, the long-term impact of drugs that employ different modes of immunomodulation is unknown and will be an important future consideration for patients and clinicians.

Despite the relatively high-risk of AID following alemtuzumab, it offers an attractive therapeutic option as a result of a unique dosing regimen, efficacy and durability of treatment as well as its ability to be incorporated safely within family planning. However, a rigorous monitoring programme together with effective patient education is essential to facilitate early identification and management of AIDs. As a result, it should be acknowledged that capacity for adherence to monitoring requirements might affect patient selection. Finally, as with all relatively novel treatments, there remains a need for continuing clinical vigilance for adverse events in clinical practice as well as long-term safety studies.
